# An Empirical Study of Economic Cycle, Air Quality, and National Health Since Reform and Opening Up

**DOI:** 10.3389/fpubh.2021.706955

**Published:** 2021-08-02

**Authors:** Kuang-Cheng Chai, Qiang Li, Xing-Li Bao, Jiawei Zhu, Xing-Xing He

**Affiliations:** Business School, Guilin University of Electronic Technology, Guilin, China

**Keywords:** business cycle, air quality, national health, reform and opening up, China

## Abstract

Since the reform and opening up of China, the economy has continued to grow, and diverse needs have generated different types and periods of economic activities. This has caused people to have an unhealthy diet, lack of exercise, irregular work and rest, lack of sleep, mental stress, high psychological pressure, long-term bad moods, and other health problems. The proportion of the sub-healthy population continues to increase and health problems are becoming increasingly prominent. Based on this, we examine the internal mechanism of the economic cycle on national health since the reform and opening up of China. For a long time, China has actively responded to the UN's call for environmental protection and proposed that “clear waters and lush mountains are invaluable assets.” Therefore, this study combines air quality in the process of environmental governance in China to investigate national health. Data from 22 provinces, five autonomous regions, and four municipalities from 2004 to 2017 are selected as research samples to examine the relationship between economic cycles, air quality, and national health for empirical testing. Studies have shown that the economic cycle is significantly correlated with national health. The better the macroeconomy, the better the health of the human body; that is, the lower the unemployment rate, the lower the mortality rate. After introducing air quality, it was found to have a significant regulating effect on the relationship between the economic cycle and national health. Our conclusions reveal that economic development is closely related to national health. China should attach great significance to the environment and air quality in the process of economic development to achieve sustainable development and create a green economy.

## Introduction

The outbreak and rapid spread of the global financial crisis in 2008 led to economic shocks and substantial recessions in many countries. For example, from 2008 to the beginning of 2014, Spain's GDP (Gross national product) and net disposable income fell by 8.1 and 9.2%, respectively (1). China's economy also shrank from a rapid growth rate of 14.16% in 2007 to a shocking 9.63% in 2008, which made a huge impact on its economy and caused operating difficulties for many companies. Under the turmoil of the financial crisis, the gap between the rich and the poor widened, and the unemployment rate remained high, leading to a further increase in income inequality and wealth gap. In addition to the economic and social losses caused by the economic recession, people have raised concerns about potential factors that threaten health. According to the World Health Organization (WHO) official data (2), in recent decades, the mortality rate from infectious diseases in China has decreased significantly (from 10 to 4%), while that caused by chronic non-communicable diseases has increased (from 79 to 89%, similar to developed countries like the United Kingdom and New Zealand). Cardiovascular diseases accounted for 43% of deaths, followed by cancer with 23%, chronic respiratory diseases with 9%, and diabetes with 2%. This demonstrates that China's health problems are still more prominent and worthy of attention.

Health is considered to be one of the important factors of human capital. It is the root of national well-being and a continuous driving force for economic development. It is undeniable that the economy is an important factor affecting health. For example, when the economy is in recession and depression, incomes decrease, consumers' purchasing power is low, and people's material and cultural needs cannot be guaranteed. For example, high wage arrears in Russia due to low oil prices may hinder healthy development (3). At the same time, labor demand declines, unemployment rate increases, the competition pressure is high, and health problems become imminent. For example, as early as 1979, Brenner (4) proposed that when the economy was in recession, unemployment caused a lack of economic security, the collapse of society and families, development of habits that are harmful to health, and even serious health problems. A recession is marked by rising unemployment and falling gross domestic product. Durkheim (5) argued that unemployment would lead to a lack of social integration among citizens, which will lead to an increase in the suicide rate. During economic expansion, the suicide rate decreases. National health status has improved significantly, and the public health response is an important part of suicide prevention during economic downturn (6).

However, a series of recent research results (7) indicate that health conditions may not necessarily deteriorate during an economic recession, and the mortality rate may decline during this period; that is, there is a “death procyclical phenomenon.” This concept contends that due to increased leisure time and the decrease in income, individual behavior will become healthier. However, recent research and analysis provide mixed evidence, and the relationship between the two is still ambiguous.

There may be an important influencing factor between the economic cycle and national health, that is, air quality. On the one hand, the WHO highlighted that environmental pollution could explain more than 30% of health fluctuations. In China, chronic respiratory diseases have accounted for 9% of the national mortality rate recently. Air quality is becoming increasingly important for population health. On the other hand, air pollution may be highly correlated with fluctuations in the economic cycle. In different economic cycle stages, energy consumption and pollutant emissions change dramatically. In the past, China used extensive economic methods with high investment, high energy consumption, and high emissions, causing a significant burden to the environment and air in the process of economic development. In recent years, China has transformed and promoted the transformation of its economic system and growth mode. The focus of the Chinese economy has shifted from “high-speed development” to “high-quality development” and the development of a green economy. At present, there is a large body of research on this aspect. Therefore, we believe that air quality is an important factor affecting the relationship between economic and national health.

In addition, with the prosperity of Bitcoin and blockchain technology, it has promoted the acceleration of the fourth industrial revolution in the financial sector (8), and introduced robo-advisors to replace traditional funds (9). The development of oil and gas is different from the past, especially under economic globalization. The economic development of various countries is also affected by other countries' economies. For example, Venezuela's oil price (OP), which has geopolitical risks, will affect inflation (INF) (10). Scholars have found that in the private investment of the seven emerging economies (E7), the increasing inflow of remittances will cause the Dutch disease, but the improvement of the quality of institutions and the combined effects of the inflow of remittances into private investment will also make the Dutch disease disappeared (11). The uncertainty of economic policies in the BRICS countries will also be impacted by crude oil prices (12). Therefore, in this context of economic globalization, based on this, this article will take China's economic development from 2004 to 2017 as the background and select Panel data from China's 22 provinces, 5 autonomous regions, and 4 municipalities directly under the Central Government are used as research samples to discuss the relationship between the economic cycle and national health from an empirical perspective rather than an empirical point of view. Air quality is used as a moderating variable to conduct in-depth research on the relationship between the economic cycle and the national health. The impact of national health, and provide suggestions for China to further develop a green economy.

## Literature Review and Research Hypothesis

In academia, opinions on the relationship between the business cycle and human health differ, and the definition of the relationship between the two is unclear.

Some researchers argue that countries with higher levels of economic development tend to have higher levels of education, better health systems, and individuals who can afford better health-related products, all of which may improve health of the population. For example, in their influential research, Pritchett and Summers (13) suggest that “the richer, the healthier.”

The cyclicality of population health has changed with the inclusion of the latest data. Tapia Granados and Ionides (14) demonstrated that the positive relationship between economic growth and population health in Sweden in the nineteenth century gradually weakened over time and was completely reversed by the end of the twentieth century. At that time, economic growth had a harmed impact on population health progress. Using data from England and Wales from 1840 to 2000, Tapia Granados (15) found a negative correlation between the growth rate of GDP and the annual growth rate of life expectancy at birth.

The impact of economic recession on health is also different. Ruhm (16) found that economic recession can improve health and reduce mortality. Some literature studies draw vague conclusions (17), including procyclical (7) and countercyclical deaths (18). For example, Lam and Piérard (19) found that the relationship between total mortality, cardiovascular mortality, and the economy became less procyclical over time and even countercyclical in recent periods in certain age groups. Deaths caused by motor vehicle accidents are cyclical.

Brenner (4) proposed that an economic recession would lead to an increase in mortality and received significant support (20–22). For example, the trend of heart disease mortality in Australia is assessed as being related to the business cycle (23). Gerdtham (24) found that during the Swedish recession, the mortality rate of men (but not women) increased significantly. During the economic recession, the development of the medical and health system was poor, the unemployment rate was high, and income was low. It was difficult for people to afford health and medical consumption. In China, as the economy develops, the unemployment rate decreases, income increases, national medical insurance is sound, and problems such as difficulty in seeing a doctor have been effectively solved. With the development of the economy, people's health will be better. This study, therefore, proposes the following hypothesis:

H1: The economic cycle is positively correlated with national health; that is, the lower the unemployment rate, the lower the mortality rate.

Since the middle of the last century, a series of problems caused by environmental pollution have attracted the attention of scholars (25). The economic development methods of countries with different income levels have brought different pressures on the environment (26). Environmental pollution causes national health problems. As a result, air quality, as a regulating variable for the economic cycle and national health, has a more obvious regulation effect. Poor air quality will harm people's physical and mental health, reduce life satisfaction, and even lead to mental illnesses such as schizophrenia and autism (27–29). A one percentage point increase in inhalable particles (PM10 and PM2.5) leads to a 0.35 percentage point increase in infant death rate (30). Mead and Brajer (31) found that the extensive use of fossil fuels caused cardiovascular and cerebrovascular diseases and increased the incidence of respiratory diseases. In the era of modern industrial economy and consumption, air pollution mainly comes from production and consumption. When the economy is in recession, production activities and consumption behaviors shrink to a certain extent. However, because the Chinese economy is in the development stage, heavy industry is the main source of air pollution. Pollution emissions are generally large, and air quality in China is a serious issue. When the economy recovered and prospered, China launched a series of green environmental protection policies, vigorously rectified heavy-polluting industries, and increased air quality. This study, therefore, proposes the following hypothesis:

H2: Air quality has a significant regulating effect on the business cycle, thereby affecting the health of the population.

## Data Source and Research Design

### Data Sources

The study selected data from 22 provinces, 5 autonomous regions, and 4 municipalities in China from 2004 to 2017 as the research samples. This study empirically tests the relationship between economic cycles, air quality, and national health. The data were mainly obtained from the China Stock Market & Accounting Research (CSMAR) database, the National Bureau of Statistics, and the Wind database.

### Variables

*National health* indicates the national health level of various provinces in China at different periods. According to the WHO's definition of health and based on previous research and common practices, *the mortality* rate is adopted as an explained variable to measure national health (8). Most studies on the relationship between previous economic cycles and mortality rates use the unemployment rate as a measure (32). Following convention, the unemployment rate is used as a variable to measure the economic cycle. *Air quality* mainly uses sulfur dioxide emissions as a regulating variable. According to previous research, demographic structure, health, and medical conditions, and income levels affect health levels. In this regard, it is measured by the child dependency ratio and medical and health improvement. Detailed descriptions of the variables are presented in Table 1.

**Table 1:** Variable definition.

**Variable type**	**Name**	**Symbol**	**Definition**
Explained variable	Mortality rate	M	Deaths per thousand
Explanatory variables	Unemployment rate	U	Total number of unemployed as a percentage of the total labor force
Control variables	Population	Population	Total population
	Urbanization rate	Urbanization	Proportion of urban population in total population (%)
	Number of medical and health institutions	Hospital	Number of medical institutions in each province
	Juvenile support rates	Child	Proportion of population aged 0–14 in total
Moderator	Sulfur dioxide	SO_2_	Sulfur dioxide emissions by Province

### Model Establishment

This article mainly uses the data of 22 provinces, 5 autonomous regions and 4 municipalities directly under the China from 2004 to 2017 as the research samples. In order to test the positive correlation between economic cycle and national health, model (1) is established:

(1) $$\begin{array}{l} M_{it}=\alpha_{0}+\alpha_{1}U_{it}+\alpha_{2}Population_{it}+\alpha_{3}\\ Urbanization_{it}+\alpha_{4}hodpital_{it}+\alpha_{5}child_{it}+\varepsilon_{it} \end{array}$$

To test the influence of air quality on the economic cycle and national health, the following model is established (2):

(2) $$\begin{array}{l} M_{it}=\beta_{0}+\beta_{1}U_{it}+\beta_{2}U_{it}\text{*}SO2_{it}+\beta_{3}Population_{it}+\\ \beta_{4}Urbanization_{it}+\beta_{5}hodpital_{it}+\beta_{6}child_{it}+\varepsilon_{it} \end{array}$$

*M*_*it*_ is an explained variable, which represents the national health of China's *i* province in year *t*. This article uses mortality as an indicator to measure the explained variable. *U*_*it*_ is an explanatory variable, which represents the economic cycle of China's province in year *t*. This article uses the unemployment rate to measure the economic cycle. *SO2*_*it*_ is a regulated variable, which represents the air quality of China's *i* province in year *t*, measured by the emission of sulfur dioxide. *Population*_*it*_, *Urbanization*_*it*_, *Hospital*_*it*_, *Child*_*it*_ are a group of control variables, which respectively represent the population, urbanization, hospitals, and juvenile support rate of *i* province in China in year *t*. These factors are factors that affect the health of the population. ε_*it*_ represents the random disturbance term. The specific explanation of the variables is shown in Table 1.

## Analysis of Empirical Results

### Descriptive Statistics

This study mainly uses the standard deviation, maximum, and minimum three dimensions to perform descriptive statistics on variables to reveal the internal laws of the data. Descriptive statistics are reported in Table 2, which also includes the mortality rate. The standard deviation is 0.715, and the difference between the maximum and minimum is 3.19, indicating that the mortality rate varies greatly in different years and provinces due to changes in time and space, and the distribution is concentrated. The unemployment and death rates are roughly the same. It can be observed that the minimum value of carbon dioxide emissions is −2.302, and the maximum value is 5.299. There is a large gap between the two values, with a standard deviation of 1.314. This indicates that the fluctuates more than in other countries, such as [include an example]. The large standard deviation of the population and the number of medical institutions means that the distribution is not concentrated. The standard deviation of the urbanization rate is 0.148, which means that the situation in China's provinces is different. The population and medical institutions are unevenly distributed, and the urban distribution is more concentrated, which also means the development of regions in China varies.

**Table 2:** Descriptive statistics.

**Variable**	**Obs**	**Mean**	**Std. Dev**.	**Min**	**Max**
M	434	5.973	0.715	4.210	7.400
U	434	3.539	0.685	1.200	6.500
Population	434	17.302	0.855	14.831	18.531
Urbanization	434	0.514	0.148	0.207	0.896
Hospital	434	9.646	0.985	7.187	11.307
Child	434	23.915	6.980	9.640	44.650
SO_2_	434	3.735	1.314	−2.302	5.299

### Correlation Analysis

The correlation test results for all the variables are reported in Table 3. The results indicate that the largest correlation coefficient is the juvenile support rate (child) and medical institutions and urbanization, which is 0.793, which indicates a (strong OR weak) correlation among the variables in the model. The correlation relationship does not cause serious multicollinearity problems. That is, multicollinearity is an interference factor in the empirical results.

**Table 3:** Correlation analysis of each variable.

		**M**	**U**	**Population**	**Urbanization**	**Hospital**	**Child**	**SO_**2**_**
M	Pearson	1						
	Sig.							
U	Pearson	0.246^**^	1					
	Sig.	0.000						
Population	Pearson	0.375^**^	0.059^*^	1				
	Sig.	0.000	0.218					
Urbanization	Pearson	−0.29^*^	−0.304^*^	0.032	1			
	Sig.	0.000	0.000	0.508				
Hospital	Pearson	0.351^**^	−0.113^*^	0.768^**^	−0.039	1		
	Sig.	0.000	0.018	0.000	0.419			
Child	Pearson	0.168^**^	0.098^*^	−0.146^**^	−0.793^**^	−0.116^*^	1	
	Sig.	0.000	0.041	0.002	0.000	0.016		
SO_2_	Pearson	0.265^**^	0.305^**^	0.741^**^	−0.046	0.520^**^	−0.09^*^	1
	Sig.	0.000	0.000	0.000	0.338	0.000	0.051	

*, ** *: Significant at the 0.1 and 0.05 level, respectively*.

### Analysis of Regression Results

It can be seen from Table 4 that in the regression of Model 1, the unemployment rate has a significant positive correlation with the death rate at the 1% level. This indicates that when the unemployment rate increases, when the economy is in recession, the death rate will increase, and there is a significant negative correlation between the economy and national health; thus, H1 was verified. Second, the coefficient of the interaction term between SO_2_ and the unemployment rate is −0.174, and it is positively correlated at the 5% level, indicating that SO_2_, that is, air quality, has a significant inhibitory and weakening effect on the relationship between unemployment and mortality, and has a significant negative regulating effect; H2 is therefore verified.

**Table 4:** Regression analysis.

**Variable**	**(1)**	**(2)**
U	0.187^***^	0.286^***^
	(3.393)	(4.431)
Population	0.229^***^	0.334^***^
	(3.395)	(4.086)
Urbanization	−0.207^***^	−0.217^***^
	(−2.694)	(−2.831)
Hospital	0.195^***^	0.188^***^
	(2.803)	(2.71)
Child	0.040	0.028
	(0.547)	(0.379)
U*SO_2_		−0.174^**^
		(−2.252)

**, *** *: Significant at the 5 and 1% levels, respectively*.

## Conclusions and Suggestions

This article selects data of 22 provinces, 5 autonomous regions, and 4 municipalities under the China from 2004 to 2017 as the research sample to explore the impact of the economic cycle on national health. It mainly uses unemployment and mortality as measurement indicators, and introduces air quality. SO_2_ is used as a measurement indicator to analyze the regulating effect of air quality. This article combines the hot spots of air quality and national health to provide strong theoretical support for the realization of green economy and circular economy in China. At the same time, national health is related to economy and air quality. The combination emphasized the importance of economy and air quality, pointed out the direction for the next development of the Chinese government and enterprises, and provided support for promoting the healthy and sustainable development of my country's economy. The specific research conclusions are as follows:

(1) There is a positive correlation between the unemployment rate and the mortality rate. The lower the unemployment rate, the lower the mortality rate. The importance of the economic cycle to the national health security is obvious, which provides more suggestions and empirical evidence for the national health field. When the unemployment rate is lower, the people have stable jobs and incomes, less competitive pressure, and less likely to cause health problems. In addition, the increase in income enables people to have sufficient funds to pay for medical expenses. The unemployment rate is an important indicator that reflects the economic development of a region. The lower the unemployment rate, the better the economic development. Since China's reform and opening up, the unemployment rate has been reduced and the economy has been developing continuously. The government has established a health system to meet the diverse medical needs of its citizens, rationally allocate medical resources, improve medical services, and continue to expand the coverage of medical insurance, in order to support the national health with a strong economy.

(2) SO_2_ has a significant negative regulation effect on unemployment and mortality. The greater the SO_2_ emissions, the weaker the promotion of unemployment on mortality, that is, the worse the air quality, the less the economic cycle's promotion of national health. With the progress of industry and human society, more pollutants are emitted, and new pollutants are generated in reaction with SO_2_, causing diseases of human organs, such as cancer, leading to an increase of death rate, affecting national health, and weaking the impact of unemployment on mortality. Based on the research on the relationship between the relatively mature business cycle and national health in the past, this paper introduces air quality as a regulating variable to construct a model. With a view to more comprehensive and in-depth analysis of the complex theory of the relationship between the two. The impact of the business cycle on national health and the regulation of air quality have been empirically verified, with higher reliability. This research not only enriches the research in this area, but also combines the current concerns about air quality and national health, and conducts research in a new direction.

Based on the above conclusions, this article proposes the following suggestions:

(1) During the expansion period of economic cyclical fluctuations, that is, when the unemployment rate is low, it is a season of increasingly active macroeconomic and market environments. The government should actively encourage employment and entrepreneurship, increase policy support and encouragement, and promote the further development of enterprises. Improve the industrial development pattern during the economic cycle expansion period, further promote the healthy development of the industrial structure, and reduce the losses caused by the transformation of the industrial structure. During the contraction period of economic cyclical fluctuations, the government must play a regulatory and protective role to avoid further economic recession and reduce the unemployment rate. China should also adhere to and use its economic foundation for a long time, build a public health system, and improve early warning and response mechanisms. Especially, after the sudden outbreak of COVID-19 in 2020, China responded quickly and controlled and contained the epidemic in a strong and effective way. Excellent results in time are worth learning from other countries.

(2) In terms of air quality, the government should increase investment, attach importance to air governance and clean air technology research and development, appropriately slow down the pace of economic development, control economic growth in an appropriate range, attach importance to the environment and air quality, and adhere to the strategy of sustainable development. Promote the development of various fields including circular economy, low-carbon economy, ecological economy, rational consumption, etc.

## Data Availability Statement

The original contributions presented in the study are included in the article/Supplementary Material, further inquiries can be directed to the corresponding author/s.

## Author Contributions

K-CC contributed to the conception of the study and directed essay writing. QL gatherded information and contributed significantly to analysis and manuscript preparation. X-LB performed the data analyses and wrote the manuscript. JZ collected and analyzed data. X-XH helped perform the analysis with constructive discussions. All authors contributed to the article and approved the submitted version.

## Conflict of Interest

The authors declare that the research was conducted in the absence of any commercial or financial relationships that could be construed as a potential conflict of interest.

## Publisher's Note

All claims expressed in this article are solely those of the authors and do not necessarily represent those of their affiliated organizations, or those of the publisher, the editors and the reviewers. Any product that may be evaluated in this article, or claim that may be made by its manufacturer, is not guaranteed or endorsed by the publisher.
